# Whole-body metabolic modelling reveals microbiome and genomic interactions on reduced urine formate levels in Alzheimer’s disease

**DOI:** 10.1038/s41598-024-55960-3

**Published:** 2024-03-13

**Authors:** Filippo Martinelli, Almut Heinken, Ann-Kristin Henning, Maria A. Ulmer, Tim Hensen, Antonio González, Matthias Arnold, Sanjay Asthana, Kathrin Budde, Corinne D. Engelman, Mehrbod Estaki, Hans-Jörgen Grabe, Margo B. Heston, Sterling Johnson, Gabi Kastenmüller, Cameron Martino, Daniel McDonald, Federico E. Rey, Ingo Kilimann, Olive Peters, Xiao Wang, Eike Jakob Spruth, Anja Schneider, Klaus Fliessbach, Jens Wiltfang, Niels Hansen, Wenzel Glanz, Katharina Buerger, Daniel Janowitz, Christoph Laske, Matthias H. Munk, Annika Spottke, Nina Roy, Matthias Nauck, Stefan Teipel, Rob Knight, Rima F. Kaddurah-Daouk, Barbara B. Bendlin, Johannes Hertel, Ines Thiele

**Affiliations:** 1 School of Medicine, University of Galway, Galway, Ireland; 2 The Ryan Institute, University of Galway, Galway, Ireland; 3 Inserm UMRS 1256 NGERE, University of Lorraine, Nancy, France; 4 Institute of Clinical Chemistry and Laboratory Medicine, University Medicine Greifswald, Greifswald, Germany; 5 Institute of Computational Biology, Helmholtz Zentrum München – German Research Center for Environmental Health, Neuherberg, Germany; 6 Department of Pediatrics, University of California San Diego, La Jolla, CA USA; 7 Department of Psychiatry and Behavioural Sciences, Duke University, Durham, NC USA; 8 Wisconsin Alzheimer’s Disease Research Center, School of Medicine and Public Health, University of Wisconsin, Madison, USA; 9 German Center of Neurodegenerative Diseases (DZNE), Rostock/Greifswald, Germany; 10 Department of Bacteriology, University of Wisconsin-Madison, Madison, WI USA; 11 German Center of Neurodegenerative Diseases (DZNE), Rostock, Germany; 12 Department of Psychosomatic Medicine, University Medicine Rostock, Rostock, Germany; 13 German Center of Neurodegenerative Diseases (DZNE), Berlin, Germany; 14 Department of Psychiatry, Charité-Universitätsmedizin Berlin, Berlin, Germany; 15 Department of Psychiatry and Psychotherapy, Charité, Berlin, Germany; 16 German Center of Neurodegenerative Diseases (DZNE), Bonn, Germany; 17 Department of Neurology, University of Bonn, Bonn, Germany; 18 German Center of Neurodegenerative Diseases (DZNE), Goettingen, Germany; 19 Department of Psychiatry and Psychotherapy, University of Goettingen, Goettingen, Germany; 20 Neurosciences and Signaling Group, Department of Medical Sciences, Institute of Biomedicine (iBiMED), University of Aveiro, Aveiro, Portugal; 21 German Center of Neurodegenerative Diseases (DZNE), Magdeburg, Germany; 22 German Center of Neurodegenerative Diseases (DZNE), Munich, Germany; 23 Institute for Stroke and Dementia Research (ISD), University Hospital, LMU Munich, Munich, Germany; 24 German Center of Neurodegenerative Diseases (DZNE), Tübingen, Germany; 25 Section for Dementia Research, Hertie Institute for Clinical Brain Research, Tübingen, Germany; 26 Section for Dementia Research, Department of Psychiatry and Psychotherapy, University of Tübingen, Tübingen, Germany; 27 DZHK (German Centre for Cardiovascular Research), Partner Site Greifswald, University Medicine, Greifswald, Germany; 28 Department of Computer Science and Engineering, University of California San Diego, La Jolla, CA USA; 29 Center for Microbiome Innovation, University of California San Diego, La Jolla, CA USA; 30 Department of Medicine, Duke University Medical Center, Durham, USA; 31 School of Microbiology, University of Galway, Galway, Ireland; 32 APC Microbiome Ireland, Cork, Ireland; 33 Department of Population Health Sciences, University of Wisconsin School of Medicine and Public Health, Madison, Wisconsin USA; 34 Shu Chien-Gene Lay Department of Engineering, University of California San Diego, La Jolla, CA USA; 35 Halıcıoğlu Data Science Institute, University of California San Diego, La Jolla, CA USA

**Keywords:** Alzheimer’s disease, Metabolic modelling, Constraint-based modelling, Microbiome, Formate, Metabolomics, Host-microbiome, Pathways, Co-metabolism, Metabolomics, Biochemistry, Systems biology, Biomarkers, Medical research, Computational biology and bioinformatics, Computational models

## Abstract

In this study, we aimed to understand the potential role of the gut microbiome in the development of Alzheimer's disease (AD). We took a multi-faceted approach to investigate this relationship. Urine metabolomics were examined in individuals with AD and controls, revealing decreased formate and fumarate concentrations in AD. Additionally, we utilised whole-genome sequencing (WGS) data obtained from a separate group of individuals with AD and controls. This information allowed us to create and investigate host-microbiome personalised whole-body metabolic models. We predicted microbial formate as well as other microbial metabolites, which could alter urine formate production in the host-microbiome personalised models. Additionally, we identified specific reactions responsible for the production of formate in the host, and interestingly, these reactions were linked to genes that have correlations with AD. This study suggests formate as a possible early AD marker and highlights genetic and microbiome contributions to its production. The reduced formate secretion and its genetic associations point to a complex connection between gut microbiota and AD. This holistic understanding might pave the way for novel diagnostic and therapeutic avenues in AD management.

## Introduction

Alzheimer’s disease (AD) is the world’s leading neurodegenerative disease and the most common cause of dementia^1,2^. Due to ageing populations and the current lack of effective treatment, AD constitutes a major socioeconomic burden in industrialised countries^3^. AD is characterised by progressing brain pathology and declining cognitive function, in addition to metabolic network failures resulting in changes in the metabolome^4^.

The human gut microbiome plays an important role in human health and well-being, performing a wide range of essential functions^5,6^. The gut microbiome affects and is affected by downstream organs, such as the liver, kidney, and brain ^7^, and is a major source of neuroprotective (e.g., indoleacrylic acid) and neurotoxic (e.g., quinolinic acid) metabolites^8^. Recently, microbial dysbiosis in AD has been reported^9,10^. Moreover, changes in gut microbial metabolites, such as primary and secondary bile acids (BAs), have been reported to correlate with mild cognitive impairment and AD dementia^11,12^. In particular, gut microbiome contributes up to 50% of formic acid human production^13^ and urinary formic acid levels have been recently suggested as non-invasive and cost-effective urinary biomarkers analysis in case of AD^14^.

Creating mechanistic computational models capable of integrating multi-omics data is essential for understanding the connections between shifts in the microbiome, changes in the metabolome, alterations in host metabolism, and the onset of diseases. The constraint-based reconstruction and analysis (COBRA) approach provides a powerful tool to better understand metabolic changes and the biochemical pathways affected by the disease^15^. COBRA relies on manually refined genome-scale metabolic reconstructions that are built bottom-up from the target organisms’ genome sequences^16^. These genome-scale reconstructions can be converted into condition-specific metabolic models through the application of constraints, such as dietary information, metagenomics, and metabolomics, thereby enabling the computation of the biologically feasible solution space of these metabolic models through widely used methods, such as flux balance analysis^17^. To meet the scale of human gut microbiomes, a resource of genome-scale reconstructions of 773 human gut microbes, called AGORA^18^, was generated and subsequently expanded in size and scope (AGORA2), now accounting for 7,302 human microbes^19^. Additionally, a large-scale reconstruction resource based on metagenomically assembled genomes has been developed accounting for nearly 250,000 genome-scale metabolic reconstructions^20^. Using these microbial reconstruction resources, comprehensive, personalised microbiome models can be constructed using microbial relative abundances derived from metagenomics data through tools, such as the Microbiome modelling toolbox^21,22^. Using personalised microbiome modelling, it is possible to provide insights and identify changes in the metabolic capabilities of gut microbiomes in disease states, including in inflammatory bowel disease^21,22^ and colorectal cancer. Such predicted metabolic capabilities could be validated against faecal metabolome data^23^. Additionally, sex-specific whole-body models (WBMs) of human metabolism have been published^24^, which represent human metabolism in an organ-resolved and anatomically accurate manner. These WBMs can be contextualised with physiological parameters, dietary input, and personalised microbiome models^24^. For the first time, these models can also be used to investigate the interaction between the host and microbiota metabolisms^24,25^and host-virus co-metabolism^26^. These simulation-derived novel hypotheses can be subsequently validated with targeted in vitro or in vivo studies.

**Figure 1:**
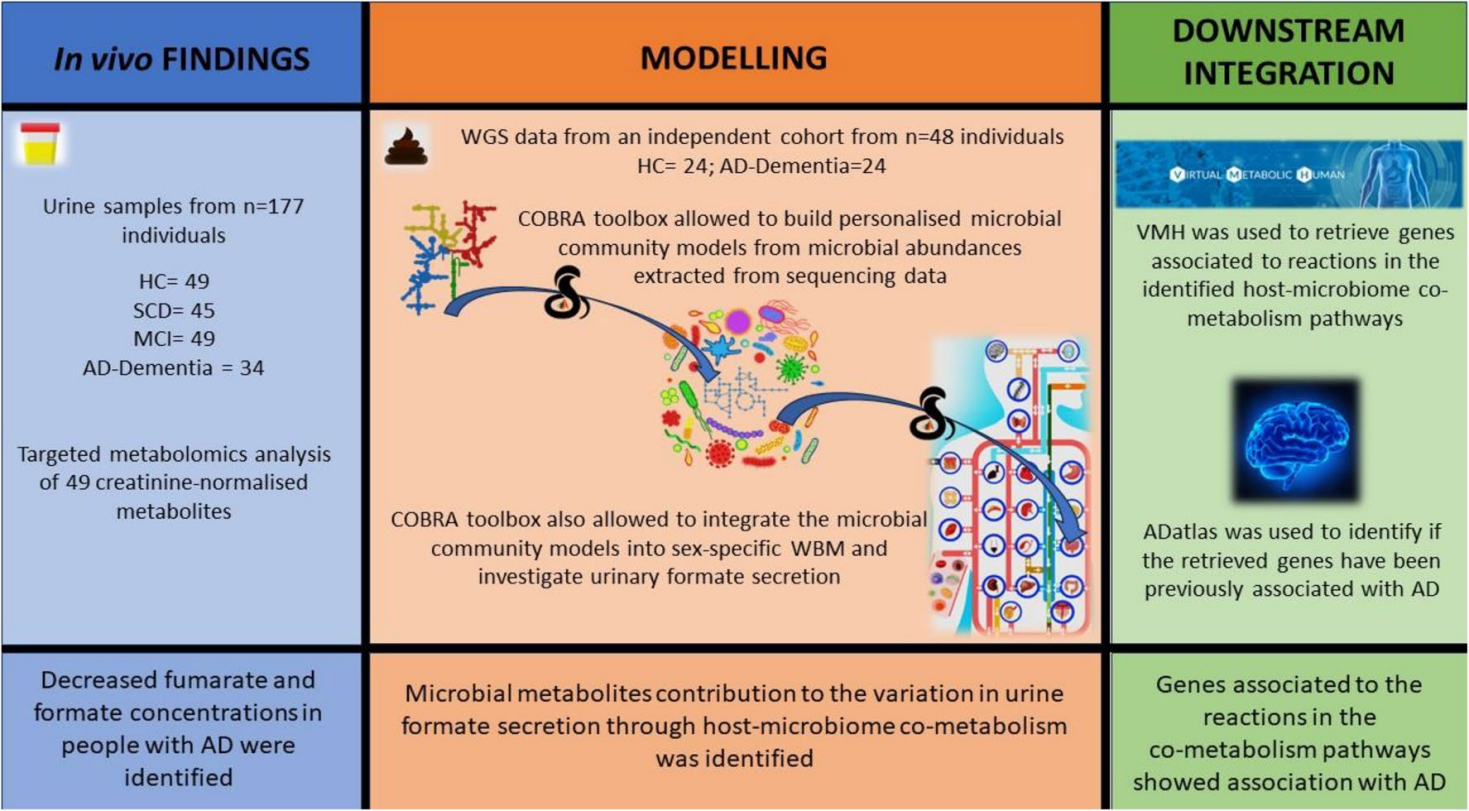
Workflow summary *HC* Healthy Controls, *SCD* Subjective Cognitive Decline, *MCI* Mild Cognitive Impairment, *AD* Alzheimer’s disease.

## Results

In this study, we aimed to characterise metabolic changes associated with cognitive decline and AD as well as to elucidate the role of the human gut microbiota in these changes. We first generated 1D-NMR urine metabolome data from individuals on the clinical AD spectrum and from cognitively healthy age-matched individuals from the Germany-based DELCODE study cohort. We identified a statistically significant decrease in formate and fumarate concentrations in people with AD dementia. To dissect potential microbial contributions, we then used WGS data generated from an independent cohort consisting of 24 participants with AD dementia and 24 cognitively unimpaired age-sex-BMI-matched controls to construct personalised host-microbiome models for each individual. We found that the formate microbial secretion was lowered in AD host-microbiome models compared to healthy control host-microbiome models. Furthermore, we found that amino acids and sugar host-microbiome co-metabolism were involved in the predicted urine formate production. The implications of these two host pathways in AD were further supported by known associations of the underlying genes with AD pathology. Taken together, our analyses point towards alterations in the microbiome’s formate secretion capacity between AD cases and controls and host-microbiome interactions on its production (Fig. 1). These findings have potential clinical relevance for understanding the interplay between the host and the microbiome in AD.

**Figure 2:**
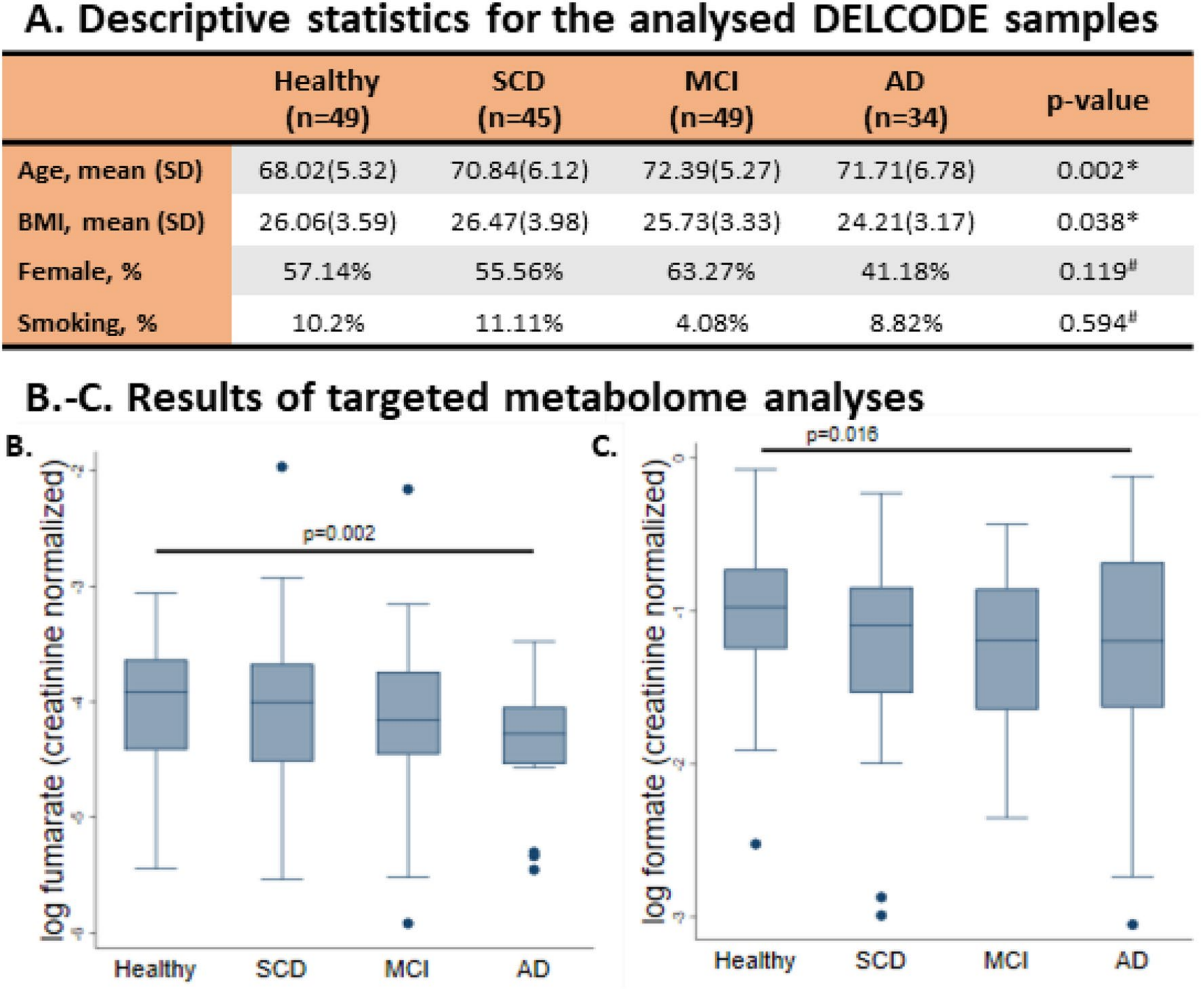
1D-NMR metabolomic data on urine samples from the DELCODE study cohort support altered metabolism in AD, including formate metabolism. (**A**) Descriptive statistics for the analysed DELCODE samples. *p-value derived from one-factorial ANOVA, ^#^*p*-value derived from Fisher’s exact test. *SCD* subjective cognitive decline, *MCI* mild cognitive impairment, *AD* Alzheimer’s disease, *BMI* body mass index. (**B**, **C**) Box plots for formate and fumarate (both creatinine normalised) over the four study groups. *p*-Values were derived from multivariable regressions adjusting for age, sex, and BMI using heteroscedastic robust standard errors. *SCD* subjective cognitive decline. *MCI* mild cognitive impairment. *AD* Alzheimer’s disease.

### Metabolomic analysis reveals decreased fumarate and formate concentrations in the urine of people with AD

To characterise altered metabolism in AD and cognitive decline, we utilised 1D-NMR urine metabolome data generated at the baseline visit from n = 177 DELCODE participants^27^ (Fig. 2A). The DELCODE cohort has four principal study groups, from which a subsample was characterised using urine metabolomics: healthy controls (n = 49), individuals with subjective cognitive decline (n = 45), mild cognitive impairment (n = 49), and AD-Dementia (n = 34). A targeted metabolomics analysis resulted in the quantification of 49 creatinine-normalised urine metabolites (Table S01). We screened all quantified metabolites with at least 50% non-zero measurements in association with the study group variable. The tricarboxylic acid (TCA) cycle intermediate fumarate was decreased in the urine among people with AD dementia (FDR < 0.05, Fig. 2B, Table S02). Furthermore, formate was also decreased in the AD dementia group, although the result was not statistically significant after correction for multiple testing (Fig. 2C, Table S02). Notably, formate and fumarate were lowered among individuals with subjective cognitive decline (SCD) as well as those with mild cognitive impairment (MCI), suggesting that altered formate and fumarate could be indicative of an early process in the aetiology of AD.

### Microbiome models showed no difference in metabolic content

**Figure 3:**
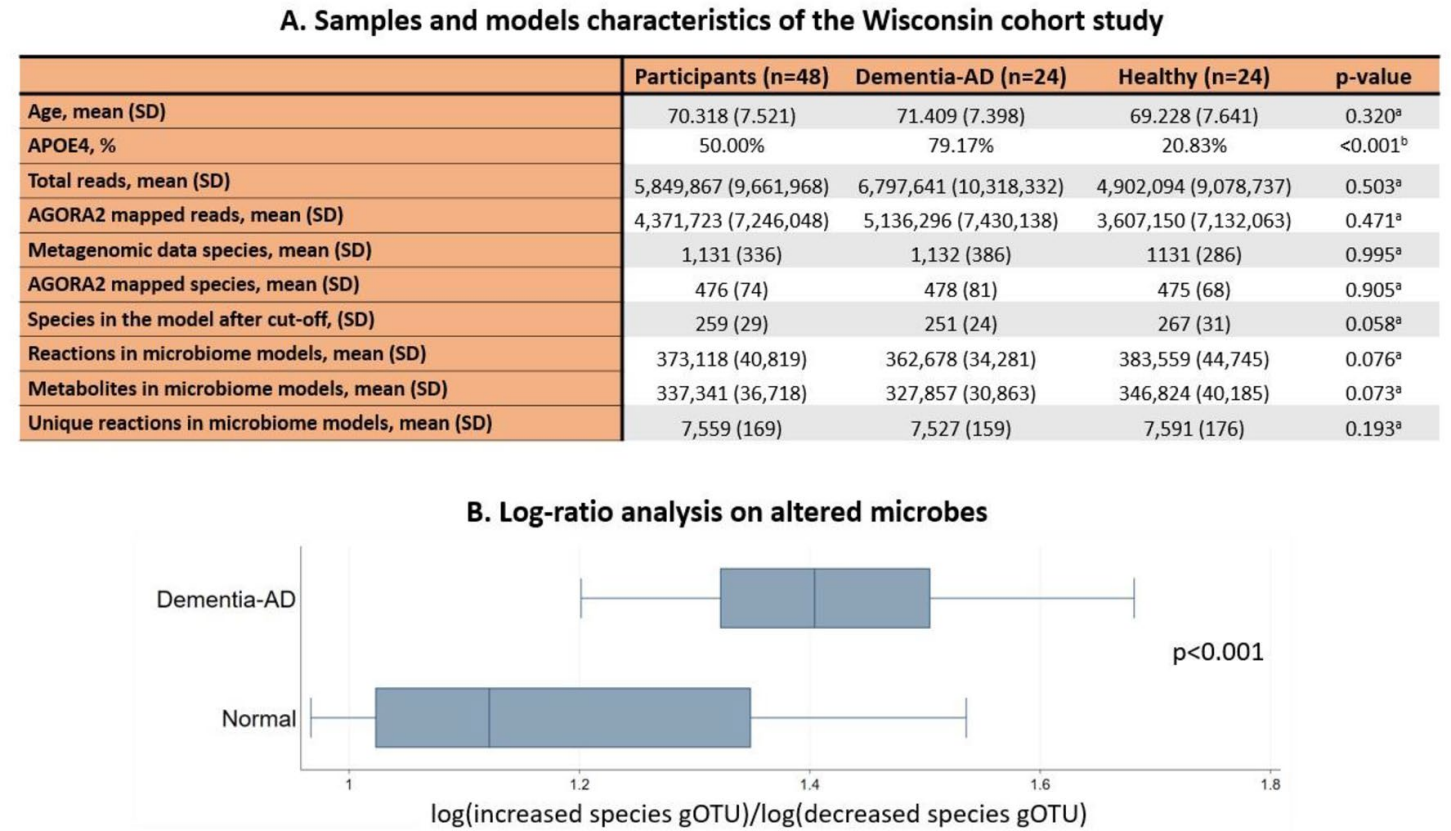
Descriptive statistics for the analysed samples and their corresponding Qiita-derived microbiome models, and diversity analysis on microbial relative abundance. (**A**) Samples and model characteristics of the Wisconsin cohort studies. *AD* Alzheimer’s disease, *SD* Standard deviation, ^a^*p*-value from Welch t-tests, ^b^p-value from Fisher’s exact test. Full results can be found in Table S03. (**B**) Boxplot of gOTU log-ratio analysis on microbes, whose models’ relative abundances were found to be altered between healthy and dementia-AD participants, *p*-value from Welch t-tests. The full results of the relative abundance analysis can be found in Table S04.

The human gut microbiome affects host metabolism and contributes up to 50% to human formate production^13^. To investigate possible microbial influences on the altered fumarate and formate levels in urine, we used personalised constrained-based models of human and microbial metabolism. As no microbiome data for the DELCODE study participants were available, we obtained stool samples from 24 subjects with dementia due to AD and 24 age- and sex-matched control participants from the Wisconsin Registry for Alzheimer’s Prevention study (WRAP) and the Wisconsin Alzheimer’s Disease Research Center cohort (WADRC)^28^. As expected, there was a higher carriage of the *APOE4* allele among people with AD dementia (Fig. 3A), the strongest genetic risk factor for late-onset AD^29,30^. For these 48 individuals, metagenomic sequence data were generated from the stool samples and analysed using the default Woltka^31^ toolkit against Web of Life (Release 1)^32^ in Qiita^33^. The median amount of reads the samples contained was 2,561,443 (interquartile range (IQR) = 2,344,948) reads. (Fig. 3A). No statistically significant differences in the number of reads could be observed between cases and controls.

To gain insight into microbiome metabolism, we used the microbial species' relative abundances to generate personalised microbiome metabolic models and we investigated their properties. Therefore, we discarded all reads that had no species taxonomic information, compared the list of identified species with the ones accounted for by the AGORA2 metabolic reconstruction resource^19^, and discarded all reads belonging to species that could not be mapped onto AGORA2. We generated pan-species microbial metabolic reconstructions consisting of the union of all metabolites and reactions present in any corresponding AGORA2 strains of the same species (“Methods”). These pan-species microbial reconstructions were then combined into one microbiome reconstruction for each sample. This AGORA2 mapping resulted in 815 different species present in at least one of the 48 samples with an average of 476 (SD = 74) microbes in each sample. After the cut-off of low abundant species (“Methods”), the sample-specific microbiome models were composed of an average of 259 (SD = 29) pan-species models, accounting for an average of 73.60% (SD = 11.92%) of the total reads in the sequencing data (Fig. 3A). The resulting 48 sample-specific microbiome models consisted, on average, of 373,118 (SD = 40,819) non-unique metabolites and 337,340 (SD = 36,718) non-unique reactions (Fig. 3A). The reaction and metabolite redundancy in the microbiome models originated from the fact that each pan-microbial reconstruction was preserved within the microbiome reconstructions. When only considering the set of unique reactions, the AD microbiome models contained on average 7,558 (SD = 169) unique reactions. No differences in the metabolic content were detected between AD and control microbiome models (Fig. 3A).

### Microbes’ abundance shifts in AD-dementia

We investigated altered microbes' relative abundances in the microbiome reconstructions and found that the relative abundances of *Turicibacter sanguinis, Hungatella hathewayi, Turicibacter sp h121, Haemophilus parainfluenzae, Ruminococcus champanellensis, Dialister succinatiphilus,* and C*oprobacillus cateniformis* were decreased and the relative abundances of A*kkermansia sp kle1797, Akkermansia sp kle1798, Parabacteroides distasonis, Bacteroides finegoldii, Collinsella tanakaei, Collinsella stercoris,* and *Bacteroides thetaiotaomicron* were increased in the AD-Dementia (participants with dementia due to AD) samples compared to the healthy controls (Table S04). Due to the compositional nature of the relative abundance data, we performed a log ratio analysis of the increased species against the decreased ones using their gOTU counts. This analysis confirmed the alteration found in the relative abundances (Fig. 3B).

### No alteration in formate urine secretion fluxes was predicted between AD dementia participants and healthy controls

Formate is a fermentation production of anaerobic bacteria in the gut and the gut microbiome metabolism can account for up to 50% of the overall formate production in the host^13^. To evaluate the possible involvement of the microbiome on formate urine excretion, we used the sex-specific, organ-resolved whole-body models of human metabolism (WBMs)^24^ and added the personalised microbiome models to the large-intestinal lumen of either the female or male WBM. Formate, which we found altered in the urine of people with AD in the DELCODE study, was part of the microbiome models and the WBMs. This metabolic overlap enabled our investigation of its direct effect on host-microbiome co-metabolism. We computed the maximum urine secretion flux and the maximum microbiome production flux for formate for all the microbiome-associated WBMs and the two sex-specific germ-free WBMs. The simulation setup of the WBMs was identical except for sex-specific host metabolism and the microbiome models. Hence, we included sex as a covariate in the subsequent statistical analyses. No statistically significant differences could be observed between healthy and AD individuals (Table S05).

### Microbial metabolites contribute to the variation in urine formate secretion through host-microbiome co-metabolism

**Figure 4:**
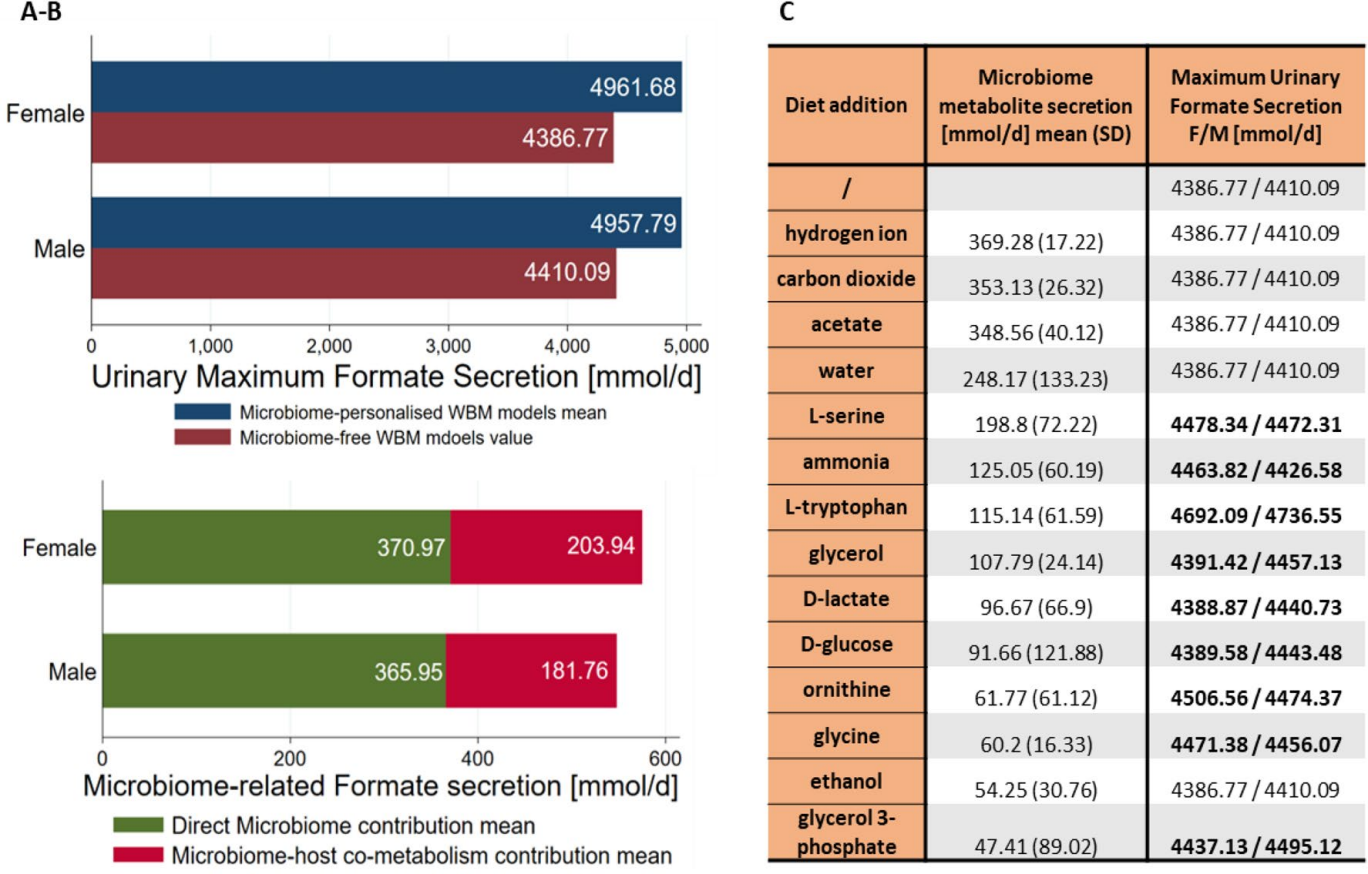
Evaluation of host-microbiome involvement in urine secretion of microbe-derived metabolites. (**A**, **B**) Breakdown of maximum sex-specific urine formate secretion highlighting the presence of microbiome-host co-metabolism. (**C**) Average metabolite microbiome secretion when the host-microbiome WBMs were interrogated for the maximum urine secretion, and urine secretions when the specific metabolites were unconstrained in the diet for the germ-free models.

To evaluate the presence and contribution of host-microbial co-metabolism in formate urinary production, we investigated the predicted differences between the formate urine fluxes of the microbiome-personalised WBMs and non-microbiome-personalised WBMs, i.e., germ-free WBMs. The difference was higher than the amount of formate secreted by the microbiomes meaning that other microbially derived metabolites must be involved in the host-microbiome co-metabolism of formate (Fig. 4A,B). To determine the main microbial metabolites that contributed to urinary formate production, we investigated the solution vector when solving the microbiome-personalised models for maximum formate urine secretion and extracted all the microbial secretion fluxes that had average flux values greater than 30 mmol/person/day (TableS06). Excluding formate, 14 metabolites were found to be highly secreted by the microbiomes. To test whether these metabolites could indeed increase the maximal urine formate secretion, we added each of the 14 metabolites individually to the diet of germ-free WBMs and solved for the maximum formate urine secretion. Nine of the 14 metabolites resulted in higher flux values for urine formate secretion compared to the average European diet without their addition and thus could be used by the host metabolism to synthesise formate (Fig. 4C). Interestingly, one of the metabolites, the ammonium ion, is not a carbon source. Nonetheless, its addition to the diet increased the formate urine secretion suggesting its involvement in formate-producing pathways. These results demonstrate that the host models metabolise microbial secretion products underlining the importance of microbial-derived metabolites as precursors of urinary formate.

### Host formate originates from the metabolism of dietary and microbial metabolites

**Figure 5:**
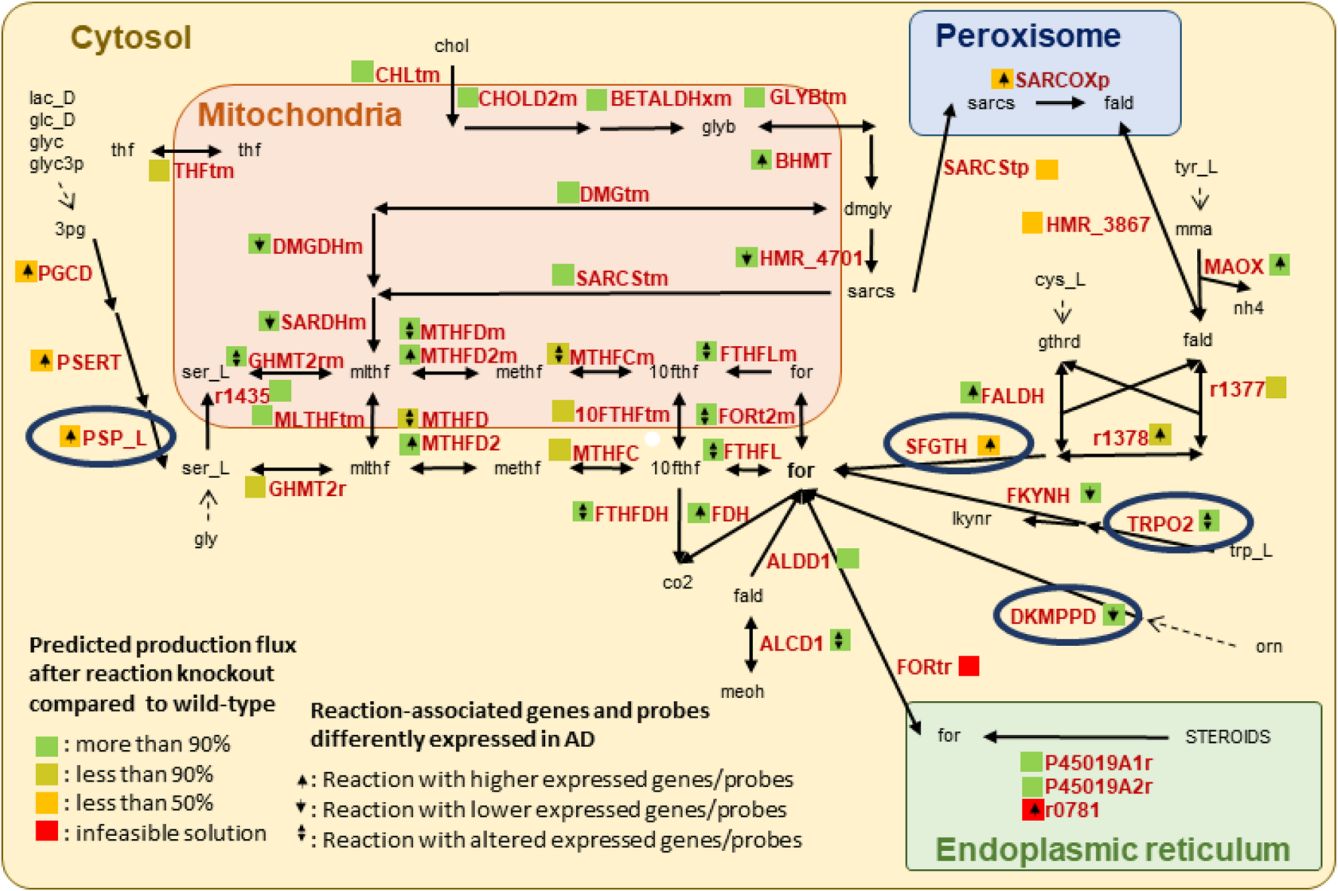
Cellular metabolism involved in the production of formate including reactions found responsible for host-microbiota co-metabolism. Dotted lines represent diet constituent involvement in the overall formate production, circled reactions when deleted together largely reduced the formate urinary production. Metabolites names (abbreviations are given in VMH IDs, www.vmh.life,^69^): *10fth* 10-formyl-THF; *3 pg* 3-phosphoglyceric acid; *chol* choline; *cys_L* L-cysteine; *dmgly* dimethylglycine; *fald* formaldehyde; *for* formate; *glc_D* D-glucose; *gly* glycine; *glyb* betaine; *glyc* glycerol; *glyc3p* glycerol-3phosphate; *gthrd* GSH; *his_L* L-histidine; *lac_D* D-lactate; *lkynr* L-kynurenine; *meoh* methanol; *methf* 5,10-methenyl-THF; *mlthf* 5,10-methylene-THF; *mma* methylamine; *orn* ornithine; *sarcs* sarcosine; *ser_L* L-serine; *thf* tetrahydrofolate; *trp_L* L-tryptophane; *tyr_L* L-tyrosine. Reactions’ names can be found in Table S07. Complete KO analysis can be found in Table S08. Differential gene expression (DEG) and differential protein abundance (DEP) analysis of association to AD-related phenotypes can be found in Table S09.

Subsequently, we aimed at elucidating the host-microbiome metabolism pathways involved in formate production. The host can produce formate through various pathways from dietary inputs^13,34^. We considered 47 reactions that have been suggested to be implicated in host formate metabolism and three transport reactions into or from the peroxisome and the endoplasmic reticulum to complete the pathways (Fig. 5, Table S07)^13^. To quantify the contribution of host metabolic reactions involved in the urine formate secretion, we deleted metabolic reactions involved in formate production in the germ-free WBM models. Overall, 17 of the 47 (36%) reaction deletions reduced the urine formate secretion flux by more than 10% (Fig. 5, Table S08). We then identified a minimal set of four reactions (VMH ID: PSP_L, Phosphoserine phosphatase; VMH ID: SFGTH, S-Formylglutathione hydrolase; VMH ID: TRPO2, L-Tryptophan: Oxygen 2,3 Oxidoreductase; and VMH ID: DKMPPD, 2,3-Diketo-5-Methylthio-1-Phosphopentane Degradation) (Fig. 5), which, when deleted together, largely reduced the urine formate secretion by the germ-free WBM models (92% reduction in male, 86% in female). These four reactions represent endpoints of multiple pathways leading eventually to formate production, either directly or indirectly via the formation of formaldehyde, which is then converted into S-formylglutathione by formaldehyde dehydrogenase (VMH ID: FALDH) and formate by the S-formylglutathione hydrolase (VMH ID: SFGTH) (Fig. 5). The four reactions were present in numerous organs in the WBM models, with all being present in the brain, pancreas, lung, and adrenal gland. Different subsets of three were also present in eight further organs (colon, stomach, liver, heart, adipocytes, gall, kidney, and thyroid gland). Notably, the four reactions were also part of the metabolic pathways of the ten microbial metabolites that we previously identified (Fig. 4C). Taken together, microbial metabolites contributed to urine formate secretion flux via host-microbiome co-metabolism involving a network of host reactions that could be mostly deactivated by the deletion of four reactions, which are broadly distributed across numerous organs.

### Reactions involved in the urine formate secretion are associated with AD

We were interested in whether any of the identified reactions and their corresponding genes have been previously associated with AD. Using the ADatlas (https://adatlas.org/)^35^ a network-based integrative resource for AD, we found that 28 of the 47 (59.5%) reactions had genes associated with AD/cognitive decline phenotypes (Fig. 5, Table S09). Nine of these 28 reactions (32%) were also predicted to alter the maximal urine formate secretion flux by more than 10% (Fig. 5). Six of these nine reactions (i.e., VMH IDs: SARCOXp, r1378, SFGTH, PGCD, PSERT, and PSP_L, full names in Table S07) were involved in the formate production through formaldehyde and 3-phosphoglycerate catabolism and positively associated with AD. Additionally, two reactions (VMH ID: FKYNH, N-formyl-L-kynurenine amidohydrolase; and VMH ID: TRPO2, L-tryptophan: oxygen 2,3-oxidoreductase) were involved in formate production through tryptophan catabolism and negatively associated with AD. Notably, reactions associated with the folate metabolism (MTHFDm, MTHFDm2, MTHFCm, MTHFD, MTHFD2) were also associated with genes differently expressed in AD, in particular with low-expression of gene *MTHFD1* and high-expression of gene *MTHFD2* (Fig. 5, Table S09). These results demonstrate that formate metabolism is altered in people with AD due to genetic variations and these genetic changes could lead to changes in formate urine secretion in addition to microbial formate production.

## Discussion

A challenge in understanding the gut microbiome’s role in health and neurological disease is linking changes in the microbiome itself and its derived metabolome to changes in host metabolism and cognitive symptoms. Here, we present a personalised metabolic modelling approach for the interrogation of the host-microbiome co-metabolism in AD. Through this framework, we used whole-body models of human metabolism to generate insights about the consequences of altered formate microbial secretion and its association with urine output through host-microbiome co-metabolism. These in silico investigations were motivated by findings of alterations in formate urine levels in AD from an independent cohort. Taken together, our analyses point towards altered gut microbiome secretion capacities and host-microbiome interactions on formate production, results that could help understand the crosstalk between host and microbiome metabolisms in AD.

The use of urine metabolomics, as a non-invasively collectable biofluid, in the context of AD and the identification of early markers of AD could be crucial to developing successful therapies^36,37^. Notably, urinary formate has been already suggested as a new potential biomarker for Alzheimer’s disease by an independent study^14^. As a final breakdown product of human and microbial metabolism, formate is typically found in human urine^38^. Levels are affected by both environmental and dietary exposures, and our results suggest that the urinary formate secretion could be a direct effect of altered host and microbial formate co-metabolism^13^. The interrogation of microbiome-personalised sex-specific WBM models, through computation of maximum formate urine secretions (Fig. 5), highlighted that microbial formate secretion capacity was lower in AD microbiomes. Our results suggested the involvement of host-microbiota co-metabolism in the overall formate urine secretion catabolising microbially derived metabolites, such as glucose, L-serine, L-glycine, L-tryptophan, L-cysteine, L-tyrosine, and ornithine. The tyrosine pathway has been repeatedly implicated in AD^39,40^ and tryptophan metabolites have been shown to regulate the cerebral activity of neprilysin, a metalloproteinase that controls the degradation and clearance of Aβ peptides in the brain^41^.

Our results further underline altered metabolism as a hallmark of AD^42,43^. In particular, the WBMs revealed the role of amino acid degradation in formate production, highlighting microbial-derived tryptophan degradation as one of the primary microbial sources of formate. Tryptophan is not only an important precursor of neurotransmitters and neuroactive metabolites, such as serotonin and kynurenine ^44^, but it also plays a role in immunoregulation ^4^. Moreover, tryptophan depletion increases cognitive deficits among people with AD^45^ and the bioavailability of metabolites in the serotonin and kynurenine pathways are altered in both the urine and serum of AD patients ^46^. The microbiome modelling implicated that microbial tryptophan production may also be reduced in AD, concurring with earlier work indicating that the microbiome contributes to human tryptophan pools ^24^. Thus, in conjunction with the WBMs, our finding of decreased formate among individuals with AD and MCI suggests alterations in tryptophan degradation in AD. These results are also in line with a recently formulated hypothesis of AD being a tryptophan metabolism-correlated disease ^47^.

Formate is also involved in different pathways and is a precursor of purine synthesis^48^. Our study also highlighted the association of formate with folate metabolism, a pathway that has been found associated with AD and DNA methylation^49^. The importance of host-microbiome formate co-metabolism is further highlighted by our examination of genes associated with AD, where we found that most of the reactions involved in formate metabolism (Fig. 5) belong to genes, whose expression was altered in AD participants compared to healthy controls. Five reactions associated with the folate metabolism (MTHFDm, MTHFDm2, MTHFCm, MTHFD, MTHFD2) were associated with genes differently expressed in AD, three reactions (VMH IDs: PGCD, PSERT, and PSP_L), which are involved in the catabolism of 3-phosphoglycerate through an alternative pathway from glycolysis with the production of L-serine, a possible precursor of formate, were associated with genes overexpressed in AD; this result could corroborate the reported reduction in glycolysis intermediate concentrations in AD participants^50^. Overall, these results suggest that formate metabolism is altered in individuals with AD also due to genetic variations, which could lead to changes in formate urine secretion in addition to microbial formate production. Notably, this inference would have not been possible without the WBM modelling, which clarified the role of the host formate metabolism. Without this additional in silico analysis, one could have falsely concluded that changes in microbial formate production, due to differences in microbiome composition, would be responsible for the reduced urine formate secretion in AD patients as measured in the metabolomic data. Since our WBM models were not further personalised using an individual’s genomic, metabolomic, or transcriptomic data, the aforementioned host genetic factors should be considered in future in silico studies, potentially increasing the validity of the in silico results regarding host urinary formate secretion.

While the COBRA modelling approach is a very valuable approach for investigating host-microbiome co-metabolism involvement in AD, certain limitations should be noted. For instance, we used a relatively small cohort of subjects and controls. Hence, our results need to be validated in larger independent cohorts with possibly urine metabolomics and WGS data from the same individuals to reduce variability. It has to be noted that discarded microbes not accounted for by the AGORA2 resource could lead to loss of metabolic capacities in the correspondent microbiome models. Additionally, the models were built on microbial relative abundance data generated from the reads count data, and results were subject to the intrinsic compositional structure of the models. Genome-scale metabolic reconstructions are also continuously updated as new experimental data and biochemical knowledge become available^51–53^. The incompleteness of the metabolic reconstructions is particularly true for gut microbes, for which only limited data are available. Computational reconstruction tools, such as DEMETER^54^, which has been used for the construction of the AGORA2 microbial reconstructions, permit the inclusion of experimental data, e.g., from BacDive^55^, during the reconstruction process. Similarly, refined genome annotations that correct missing and mis-annotations should be performed to minimise the errors in the reconstruction, and thus increase the fidelity of the predictions. Such reannotation has been done for most of the microbes in AGORA2. Moreover, an inherent limitation of the COBRA approach is that it assumes the biological system to be in a steady-state condition, thereby ignoring the dynamic nature of microbial communities. Notably, the predicted secretion capacities obtained through microbiome modelling are not confounded by different factors, such as age and sex, improving the identification of secretion-microbial correlations, since they are derived from deterministic modelling rather than inferred from statistical dependence patterns of observational data. Additionally, while this study highlighted the role of gut microbes and host metabolism and genetics, differences between lifestyle factors (e.g., diet and exercise) and medications are also likely to contribute to changes in host-microbiome co-metabolism, urine metabolome, and AD pathology.

In conclusion, in this study, we combined omics data with COBRA modelling on the level of the microbiome and the whole human supra-organism and highlighted the role of microbiome-host interplay on formate-producing pathways. In particular, the microbiome’s role in linking aminoacidic and glucose metabolism with formate, a possible early marker for AD, could be of clinical importance, potentially contributing to the AD phenotype. The underlying mechanism suggested by our model, that both gut microbes and host genetics contribute to an altered formate metabolism in AD, needs to be assessed with more targeted validation studies. Our study delivers proof of the concept of personalised whole-body modelling in the context of a complex human disease. As such, the paradigm has demonstrated promise in uncovering host-microbiome co-metabolism involving biomarkers found in metabolomic studies validating or suggesting pathology hypotheses.

## Methods

This study did not generate new unique reagents.

### Statistical analyses

For descriptive statistics, metric variables were expressed in means and standard deviations, while categorical variables were described by proportions. All p-values were reported as two-tailed. The statistical analyses were performed with STATA 16.1/MP (STATA Inc., College Station, Texas, USA).

### DELCODE cohort

#### Study sample

We used an interim data-freeze from the DELCODE study conducted by the Deutsches Zentrum für Neurodegenerative Erkrankungen (DZNE)^27^. Note that the DELCODE study did not include individuals with a current major depressive episode, major psychiatric disorders, neurological diseases other than AD, or unstable medical conditions^27^. For our study, we also excluded individuals who had no urine samples available. The resulting sub-cohort consisted of 49 healthy controls, 45 cases with subjective cognitive decline (n = 45), 49 cases with mild cognitive impairment, and 34 cases with AD dementia (Fig. 2A). We defined subjective cognitive decline (SCD) as a constant self-perceived cognitive decline without observation of any objective cognitive impairment as measured by the CERAD test battery, persisting at least for six months and being unrelated to an episodic event^56^. The National Institute on Aging-Alzheimer’s Association (NIA-AA) workgroup guidelines^57,58^ were used to define the core clinical criteria for MCI and dementia AD-related. The control subjects showed no objective cognitive impairment in cognitive tests, had no history of neurological or psychiatric disease, and did not report a self-perceived cognitive decline. Informed consent was provided by all participants or their representatives. The study protocol was approved by the local institutional review boards and ethics committees of the participating centres and the study was conducted in accord with the Helsinki Declaration of 1975.

#### ^1^H-NMR measurements

The urine samples were handled and prepared as in^59^. The urine samples from 185 individuals were measured at the University Medicine Greifswald on a Bruker AVANCE-II 600 NMR spectrometer operated by TOPSPIN 3.2 software (both Bruker Biospin, Rheinstetten, Germany). The spectrometer was equipped with a 5-mm z-gradient probe and an automated tuning and matching (ATMA) unit (both Bruker Biospin, Rheinstetten, Germany). Specimens were automatically delivered to the spectrometer via SampleJet (Bruker Biospin, Rheinstetten, Germany) into standard 5 mm NMR tubes. The acquisition temperature was set to 300°K. A standard one-dimensional ^1^H-NMR pulse sequence with suppression of the water peak (NOESYPREAST) was used^60^. Urine samples of four individuals could not be processed at all due to too little biomaterial available for ^1^H-NMR measurements. After processing the raw spectra, 50 metabolites were quantified using the Bruker Suite B.I.Quant-UR b™ for targeted analyses. To account for dilution, two different normalisation approaches were performed: (1) creatinine normalisation and (2) PQN normalisation. Different normalisation approaches have been shown to have different advantages as well as disadvantages such that it is recommended to use multiple approaches and to compare results across normalisation techniques^61^. For quality check purposes, creatinine was measured via an enzymatic standard kit to compare ^1^H-NMR-derived creatinine measurements with standard creatinine measurements. Four further observations were excluded for strong differences between enzymatic and ^1^H-NMR creatinine measurements. The remaining observations showed a correlation > 0.95 between the two types of creatinine measurements. In the end, n = 177 spectra were included in statistical analyses.

#### Descriptive sample statistics of targeted metabolome data

Metadata and urine samples for 177 individuals belonging to four study groups were available for analysis (Fig. 2A). As described above, eight individuals were excluded from analyses, thus the final sample, for which urine metabolome data was available, comprised 177 individuals. Figure 2A lists the descriptive statistics for basic sample characteristics. Importantly, the four study groups (healthy, subjective memory impairment, MCI, AD) were not balanced for age and body mass index (BMI, Fig. 2A). Therefore, all statistical metabolome analyses accounted for age and BMI differences by including age and BMI as covariates in the statistical modelling.

#### Statistical analyses of the NMR metabolome data

Descriptive statistics were derived for 49 metabolites after creatinine normalisation. Of those 49 metabolites, only 16 metabolites had non-zero urinary concentration measurements in more than 50% of the samples. Importantly, a zero measurement in the context of NMR measurement does not necessarily mean that the metabolite was below the limit of detection. The signal of the correspondent metabolite may also have been clouded by other metabolites or may have been shifted due to pH differences in ion concentrations, hence, we treated zero measurements as missing values. We screened all metabolites fulfilling the 50% criteria via multivariable linear regressions including age, sex, and BMI as covariates. Log-transformed, creatinine-normalised metabolite concentrations were used as the dependent variables, and the study group variables (categorical) were the predictors of interest. We performed the global test using the Wald-test method to investigate 1. whether the study group variable contributed overall to the statistical model and 2. whether there were specific differences between the AD group and the healthy controls. For correction of multiple testing, the false discovery rate was utilised.

### WISCONSIN cohort

#### Faecal samples

Our study utilised data from the Wisconsin Alzheimer’s Disease Research Center (WADRC) and the Wisconsin Registry for Alzheimer's Prevention (WRAP) studies. Participants with AD-Dementia were recruited from the WADRC, while cognitively unimpaired participants were recruited from the WADRC and the WRAP studies. Data on age, sex, BMI, *APOE* genotype, diagnosis, and cognitive test results were collected^62,63^. Our study used a subset of the cohort comprising 48 individuals (24 AD-Dementia cases and 24 sex and age-matched healthy controls). Covariates were compared against the clinical diagnosis using Fisher’s exact test for *APOE4* and sex, and Welch’s t-test for age and BMI. The study protocol was approved by The University of Wisconsin Health Sciences Institutional Review Board.

#### Sequencing and processing of metagenomic data

The faecal samples were sequenced and then processed through Qiita^33^ using the default workflow for metagenomics data. In short, the raw files were loaded in multiple preparations to represent the multiple runs and processed with default parameters; then the raw sequencing data were demultiplexed and trimmed at 150 bases. Adapter removal was carried out using fastp^64^ and human reads were filtered using minimap2^65^. Genomic OTUs (gOTUs) were generated using the Woltka Toolkit^31^ by aligning reads to the Web of Life (Release 1) reference genome database^32^ using bowtie2^66^.

#### Construction of personalised microbiome metabolic models

First, we assigned taxonomy to gOTUs using the Web of Life database, and we summed together the gOTUs with matching taxonomical information. Reads with no specific species information or associated to species not accounted by our database of genome-scale metabolic microbial reconstructions (AGORA2)^19^ were discarded. The remaining reads were normalised such that the sum of all reads was one, thereby obtaining the relative abundances of each microbe in the sample. The obtained relative abundances were then mapped to 1,742 AGORA2 pan-species reconstructions, the union of reactions and metabolites of each strain-specific reconstruction of one species. Then, sample-specific microbiome models were derived using the mgPipe module of the Microbiome Modelling Toolbox^21,22^. The process involves integrating various microbial reconstructions by introducing additional reactions into each microbial reconstruction to allow the transfer of metabolites between the extracellular space of each microbe and a shared lumen compartment^67^. This lumen compartment is interconnected with both dietary and faecal compartments, allowing for the absorption and excretion of metabolites to and from the surrounding environment. Within each microbial community model, adjustments are made to the community biomass reaction based on the provided relative abundance data. Further constraints were then applied to couple each microbial reaction to its corresponding biomass reaction. These ‘coupling constraints’ ensure that flux through a pan-genome model within the microbiome model was only non-zero if the corresponding biomass reaction carried a non-zero flux. The coupling factor was arbitrarily chosen to be 400^67,68^. We tested for differences in measures of metabolic content in the microbiome models between cases and controls using linear regressions correcting for the presence of the *APOE4* allele.

#### Species abundances evaluation

To investigate differences in the AGORA2-covered microbial abundances between cases and controls, we fitted fractional regressions using the relative microbial abundances as the response variable and the health status as an independent one correcting for categorical *APOE* genotype, while we excluded all the microbes present in less than 50% of the models to avoid statistical artefacts. Alteration in relative species abundances was checked using log-ratio analysis, after calculating the sum of the gOTUs for the increased species and decreased species, the ratio was calculated and a t-test was run between groups.

#### Diet constraints

An average European Diet^69^, supplemented with bile acids (cholic acid and chenodeoxycholic acid), was applied to further constrain each microbiome model and to convert them into condition-specific models (Table S11). The diet constraints were defined to be in mmol/person/day. We integrated the uptake fluxes values defined by the diet with all the microbiome models using the Microbiome Modelling Toolbox^21^ implemented in the COBRA toolbox^70^, and we ensured that all the pan-species models could grow under the defined diet.

#### Microbiome model simulations

Each microbiome model under the given condition-specific constraints was interrogated using flux variability analysis (FVA)^71^ to obtain the corresponding maximum net secretion capacity for all the model’s secretion reactions, FVA solves maximisation problems with the interested reactions secretion fluxes as objective subject to different constraints to specify the feasible region: predetermined upper and lower bound of reactions fluxes stated in the model, diet fluxes, and mass balance in form of (S.v = dv/dt = 0, whereas is the stoichiometric matric with metabolites as rows and reactions as columns and v is the flux vector). For each metabolite secretion capacity, zero values meant that the model could not secrete the corresponding metabolite, while a positive value corresponded to the microbiome model’s capacity of secreting the metabolite, under the given simulation constraints. All simulations were performed in MATLAB (Mathworks, Inc.) version R2018b using IBM CPLEX (IBM, Inc.) as a linear programming solver and the COBRA Toolbox v3^70^ and the Microbiome Modelling Toolbox^22^.

#### Interrogation of whole-body metabolic (WBM) personalised models

We added the individual microbiome models to the large intestinal lumen of either female or male organ-resolved, whole-body models of human metabolism (WBMs), as appropriate, using the PSCM toolbox^24^. We computed the maximum urine secretion fluxes of metabolites, which were significant in the previous analyses and could be produced by the microbiome-associated WBM under an average European diet^69^. The male and female WBMs, which correspond to a reference man and woman^24^, respectively, were not further personalised as the sub-cohort was age and BMI matched between healthy and AD individuals. The maximum urine secretion rate for each microbiome-associated WBM was calculated by choosing the corresponding urine metabolite exchange reaction (e.g., ‘EX_for[u]’ for formate) as an objective function and maximising for this reaction, using the PSCM toolbox^24^. Maximum urine secretion rates for the investigated metabolites were also calculated for non-personalised, germ-free, male and female WBMs. The maximum microbial secretion fluxes were calculated by choosing the corresponding microbial metabolite exchange reaction between the microbial lumen [luM] and large intestinal lumen [luLI] and solving the models minimising the reaction.

#### Differences in secretion fluxes between AD cases and controls

To investigate differences in reaction fluxes between cases and controls, we fitted linear regression using the fluxes as the response variable, the health status as an independent variable correcting for categorical *APOE* genotype, and sex being the WBM models sex-specific.

#### Investigation of host-microbiota co-metabolism

To investigate the involvement of host-microbiota co-metabolism in formate production, we considered the difference in secretion of formate between the personalised and the germ-free models and we compared these differences with the community microbial secretion of the same metabolite to evaluate the amount of urine secretion directly correlated to the microbial secretion and the amount correlated to host-microbiota co-metabolism.

We confirmed the capacity of the whole-body models to produce formate from other microbial metabolites by adding individually to the diet metabolites that can be secreted by the microbiome. All metabolites were added to the diet setting their potential maximum intake to 300 mmol/d. These implemented diets were tested for maximum urine formate production on the germ-free models to evaluate changes in the previous baseline value.

#### Genetic involvement in host formate production pathways in AD

We considered all the reactions known from the literature^13^ to be involved in the formate metabolism including the ones we found responsible for the host-microbiota co-metabolism. We retrieved all the genes associated with these reactions from the Virtual Metabolic Human database (VMH)^69^, which houses the generic human metabolic reconstruction, Recon3D^52^, and then we investigated for associations between genes and health status using the AD Atlas^35^ a network-based integrative resource for AD. The AD Atlas integrates multi-omics data from large-scale population-based and AD case–control studies, enabling users to annotate genes, metabolites, or phenotypes of interest in an AD-related context. In particular, differential expression genetic (DEG) data had been integrated in AD atlas from 2,114 postmortem human brain samples^72^. In our study, the AD atlas was queried using the genes identified to be involved in formate metabolism, retrieving information on brain-region-specific differential gene expression and differential protein abundance in AD, as well as genetic associations to AD-related phenotypes inferred from large-scale genome-wide association studies (GWAS). A gene-wise significance threshold was applied to filter GWAS results, as implemented by the AD Atlas^35^.

## Supplementary Information

Supplementary Information.

## Data Availability

The data supporting the conclusions of this study are accessible; however, constraints are imposed on their public availability. Notwithstanding, these data can be procured from the authors upon the submission of a reasonable request. Data from the DELCODE cohort will be made available on request to Johannes Hertel (Johannes.Hertel@med.uni-greifswald.de). Data from the WISCONSIN cohort will be made available on request to Barbara B. Bendlin (bbb@medicine.wisc.edu).
