# Author Correction: Whole-body metabolic modelling reveals microbiome and genomic interactions on reduced urine formate levels in Alzheimer’s disease

**DOI:** 10.1038/s41598-024-78228-2

**Published:** 2024-11-12

**Authors:** Filippo Martinelli, Almut Heinken, Ann-Kristin Henning, Maria A. Ulmer, Tim Hensen, Antonio González, Matthias Arnold, Sanjay Asthana, Kathrin Budde, Corinne D. Engelman, Mehrbod Estaki, Hans-Jörgen Grabe, Margo B. Heston, Sterling Johnson, Gabi Kastenmüller, Cameron Martino, Daniel McDonald, Federico E. Rey, Ingo Kilimann, Olive Peters, Xiao Wang, Eike Jakob Spruth, Anja Schneider, Klaus Fliessbach, Jens Wiltfang, Niels Hansen, WenzelGlanz, Katharina Buerger, Daniel Janowitz, Christoph Laske, Matthias H. Munk, Annika Spottke, Nina Roy, Matthias Nauck, Stefan Teipel, Rob Knight, Rima F. Kaddurah-Daouk, Barbara B. Bendlin, Johannes Hertel, Ines Thiele

**Affiliations:** 1 School of Medicine, University of Galway, Galway, Ireland; 2 The Ryan Institute, University of Galway, Galway, Ireland; 3 Inserm UMRS 1256 NGERE, University of Lorraine, Nancy, France; 4 Institute of Clinical Chemistry and Laboratory Medicine, University Medicine Greifswald, Greifswald, Germany; 5 Institute of Computational Biology, Helmholtz Zentrum München – German Research Center for Environmental Health, Neuherberg, Germany; 6 Department of Pediatrics, University of California San Diego, La Jolla, CA USA; 7 Department of Psychiatry and Behavioural Sciences, Duke University, Durham, NC USA; 8 Wisconsin Alzheimer’s Disease Research Center, School of Medicine and Public Health, University of Wisconsin, Madison, USA; 9 German Center of Neurodegenerative Diseases (DZNE), Rostock/Greifswald, Germany; 10 Department of Bacteriology, University of Wisconsin-Madison, Madison, WI USA; 11 German Center of Neurodegenerative Diseases (DZNE), Rostock, Germany; 12 Department of Psychosomatic Medicine, University Medicine Rostock, Rostock, Germany; 13 German Center of Neurodegenerative Diseases (DZNE), Berlin, Germany; 14 Department of Psychiatry, Charité-Universitätsmedizin Berlin, Berlin, Germany; 15 Department of Psychiatry and Psychotherapy, Charité, Berlin, Germany; 16 German Center of Neurodegenerative Diseases (DZNE), Bonn, Germany; 17 Department of Neurology, University of Bonn, Bonn, Germany; 18 German Center of Neurodegenerative Diseases (DZNE), Goettingen, Germany; 19 Department of Psychiatry and Psychotherapy, University of Goettingen, Goettingen, Germany; 20 Neurosciences and Signaling Group, Department of Medical Sciences, Institute of Biomedicine (iBiMED), University of Aveiro, Aveiro, Portugal; 21 German Center of Neurodegenerative Diseases (DZNE), Magdeburg, Germany; 22 German Center of Neurodegenerative Diseases (DZNE), Munich, Germany; 23 Institute for Stroke and Dementia Research (ISD), University Hospital, LMU Munich, Munich, Germany; 24 German Center of Neurodegenerative Diseases (DZNE), Tübingen, Germany; 25 Section for Dementia Research, Hertie Institute for Clinical Brain Research, Tübingen, Germany; 26 Section for Dementia Research, Department of Psychiatry and Psychotherapy, University of Tübingen, Tübingen, Germany; 27 DZHK (German Centre for Cardiovascular Research), Partner Site Greifswald, University Medicine, Greifswald, Germany; 28 Department of Computer Science and Engineering, University of California San Diego, La Jolla, CA USA; 29 Center for Microbiome Innovation, University of California San Diego, La Jolla, CA USA; 30 Department of Medicine, Duke University Medical Center, Durham, USA; 31 School of Microbiology, University of Galway, Galway, Ireland; 32 APC Microbiome Ireland, Cork, Ireland; 33 Department of Population Health Sciences, University of Wisconsin School of Medicine and Public Health, Madison, WI USA; 34 Shu Chien-Gene Lay Department of Engineering, University of California San Diego, La Jolla, CA USA; 35 Halıcıoğlu Data Science Institute, University of California San Diego, La Jolla, CA USA

Correction to: *Scientific Reports* 10.1038/s41598-024-55960-3, published online 13 March 2024.

The original version of this Article contained an error in the Abstract.

“Notably, AD individuals displayed diminished formate microbial secretion in these models.”

now reads:

“We predicted microbial formate as well as other microbial metabolites, which could alter urine formate production in the host-microbiome personalised models.”

The original Article has been corrected.

